# Safety and Efficacy of Spironolactone in Dialysis-Dependent Patients: Meta-Analysis of Randomized Controlled Trials

**DOI:** 10.3389/fmed.2022.828189

**Published:** 2022-03-17

**Authors:** Jing Liu, WanYu Jia, Chen Yu

**Affiliations:** ^1^Department of Nephrology, Tongji Hospital, Tongji University School of Medicine, Shanghai, China; ^2^Department of Pediatrics, Clinical Center of Pediatric Nephrology of Henan Province, The First Affiliated Hospital of Zhengzhou University, Zhengzhou, China

**Keywords:** dialysis, end-stage renal disease, mineralocorticoid receptor antagonists, spironolactone, meta-analysis

## Abstract

**Background:**

Patients with end-stage renal disease (ESRD) are characterized with high risk of heart failure. Although mineralocorticoid receptor antagonists have beneficial effect on relieving cardiac fibrosis and, thus, reduce the incidence of cardiovascular disease and cardiac death, the therapeutic benefits and adverse effects are still controversial. We conducted a meta-analysis to measure the safety and efficacy of spironolactone in patients undergoing dialysis.

**Methods:**

A systematic search for randomized controlled trials (RCTs) was performed in PubMed, Embase, and Cochrane databases. Primary outcomes included changes in all-cause mortality (ACM), serum potassium concentration, incidence of hyperkalemia and gynecomastia (GYN). Secondary outcomes included changes in blood pressure (BP), left ventricular mass index (LVMI) and left ventricular ejection fraction (LVEF). Subgroup analysis and sensitivity analysis were further conducted. This research was registered with PROSPERO (International Prospective Register of Systematic Reviews; No. CRD42021287493).

**Results:**

Fifteen RCTs with 1,258 patients were enrolled in this pooled-analysis. Spironolactone treatment significantly decreased ACM (RR = 0.42, *P* < 0.0001), CCV (RR = 0.54, *P* = 0.008) and LVMI (MD = −6.28, *P* = 0.002), also increased occurrence of GYN (RR = 4.36, *P* = 0.0005). However, LVEF (MD = 2.63, *P* = 0.05), systolic BP (MD = −4.61, *P* = 0.14) and diastolic BP (MD = −0.12, *P* = 0.94) did not change between two groups after treatment. Although serum potassium concentration was increased (MD = 0.22, *P* < 0.0001) after spironolactone supplement, the risk of hyperkalemia remained unchanged (RR = 1.21, *P* = 0.31). Further subgroup analysis found more obvious advantageous as well as disadvantageous effects in Asian subjects than European or American ones. Also, with more than 9 months of treatment duration, patients achieved more favorable influence than shorter duration.

**Conclusions:**

These results highlight the therapeutic effects of spironolactone on cardiovascular indexes, including ACM, CCV, and LVMI. However, the unignorable increase of GYN incidence and serum potassium level indicate that close monitor in dialysis-dependent patients, especially Asian patients, is essential.

## Introduction

Heart failure, which often occurs in patients with chronic kidney disease (CKD), may contribute to high cardiovascular morbidity and mortality (1, 2). Among all causes of death in patients undergoing dialysis, sudden cardiac death is the leading one, accounting for 25% of all-cause mortality (ACM) (3). Hypertension and left ventricular hypertrophy, which is directly associated with the risk for sudden cardiac death and ACM, occur in more than 70% patients with long-term dialysis (4, 5).

Aldosterone has been implicated as an important factor to keep cardiovascular homeostasis. As a pleiotropic hormone, aldosterone also can regulate various tissues, such as heart, kidney and liver, through activating the mineralocorticoid receptors (MRs) (6). In the presence of impaired renal function, the renin-angiotensin-aldosterone system (RAAS) are always activated abnormally, mediating high blood pressure and cardiac fibrosis (7). Former researches proved that utilization of mineralocorticoid receptor antagonists (MRA) can mitigate the deleterious effects on cardiovascular system and thus, improving the prognosis of patients with end-stage renal disease (ESRD) (8).

The role of MRAs therapy as a neurohormonal antagonist has been studied by prior studies, however, had various outcomes. Flevari et al. (9) found significant increased sodium potassium level and decreased blood pressure after MRAs treatment, while Lin et al. (10) and Gross et al. (11) suggested unchanged serum potassium and blood pressure, respectively. Previous meta-analyses have been limited by small number of clinical trials (12) or results for single system (13). Thus, spironolactone is not widely understood in subjects undergoing dialysis. These differences in adverse effect and efficacy prompted us to conduct a meta-analysis to determine the changes in dialysis-dependent patients after spironolactone supplement. Also, we will further explore the effect of some factors (e.g., country, dosage, intervention duration) on the results through subgroup analysis.

## Methods

This meta-analysis was conducted in accordance with the preferred reporting items for systematic reviews and meta-analyses (PRISMA) guideline, and was registered with PROSPERO (International Prospective Register of Systematic Reviews; No. CRD42021287493).

### Search Strategy and Data Sources

Literature published up to October 2021 in PubMed, Embase, and Cochrane databases, without time or language restriction, were searched. The search strategies are provided in Appendix A. Also, the reference lists of review articles and original studies were manually searched for additional eligible reports.

### Selection Criteria

Studies were considered to be eligible if they met the following criteria: (1) randomized-control study on humans; (2) dialysis patients (3) patients in the intervention group, were treated with spironolactone, while the control subjects received placebo or standard treatment. Exclusion criteria were: (1) compared different dosages of spironolactone; (2) failed to provide data on outcomes of interest: occurrence of adverse events (ACM, hyperkalemia and gynecomastia [GYN]) and cardiovascular benefits (including incidence of cardiocerebrovascular diseases [CCV], left ventricular mass index [LVMI], left ventricular ejection fraction [LVEF], systolic blood pressure [SBP] and diastolic blood pressure [DBP]).

### Data Extraction and Quality Assessment

The data extraction from each studies includes study characteristics (year of publication, country, randomization method, type of study, sample size, duration and follow-up period) and patient characteristics (age, sex, and dialysis type).

### Statistical Analyses

Review Manager (RevMan, version 5.4; the Nordic Cochrane Center, the Cochrane Collaboration, Copenhagen, Denmark) and Stata/SE (version 15.1; StataCorp LP, College Station, TX) were used for the analysis. Two authors extracted raw data from individual studies and then calculated pooled risk ratios (RRs) for dichotomous outcomes and mean differences (MDs) for continuous ones, and corresponding 95% confidence intervals (95% CIs) for each outcomes. The outcomes are presented as SMD if continuous indexes were measured in different methods. For research with more than one intervention groups, we split the shared control group into several groups with smaller sample size, and thus, included two or more comparisons (14). A fixed-effects model was used to perform meta-analysis, and a random-effects model was applied when severe heterogeneity was present. Heterogeneity of included studies was quantified by *Q* test and *I*^2^ statistic. High heterogeneity was defined as *p* < 0.1 for *Q* statistic or *I*^2^ > 50%. To identify the source of heterogeneity, subgroup analysis was further conducted, according to country, dosage, and length of follow-up. Besides, two reviewers performed additional sensitivity analyses to explore the impact of a single article on the results. Cochrane Collaboration methodology (14) was used to assess included studies for bias.

## Results

### Search Results

**Figure 1:**
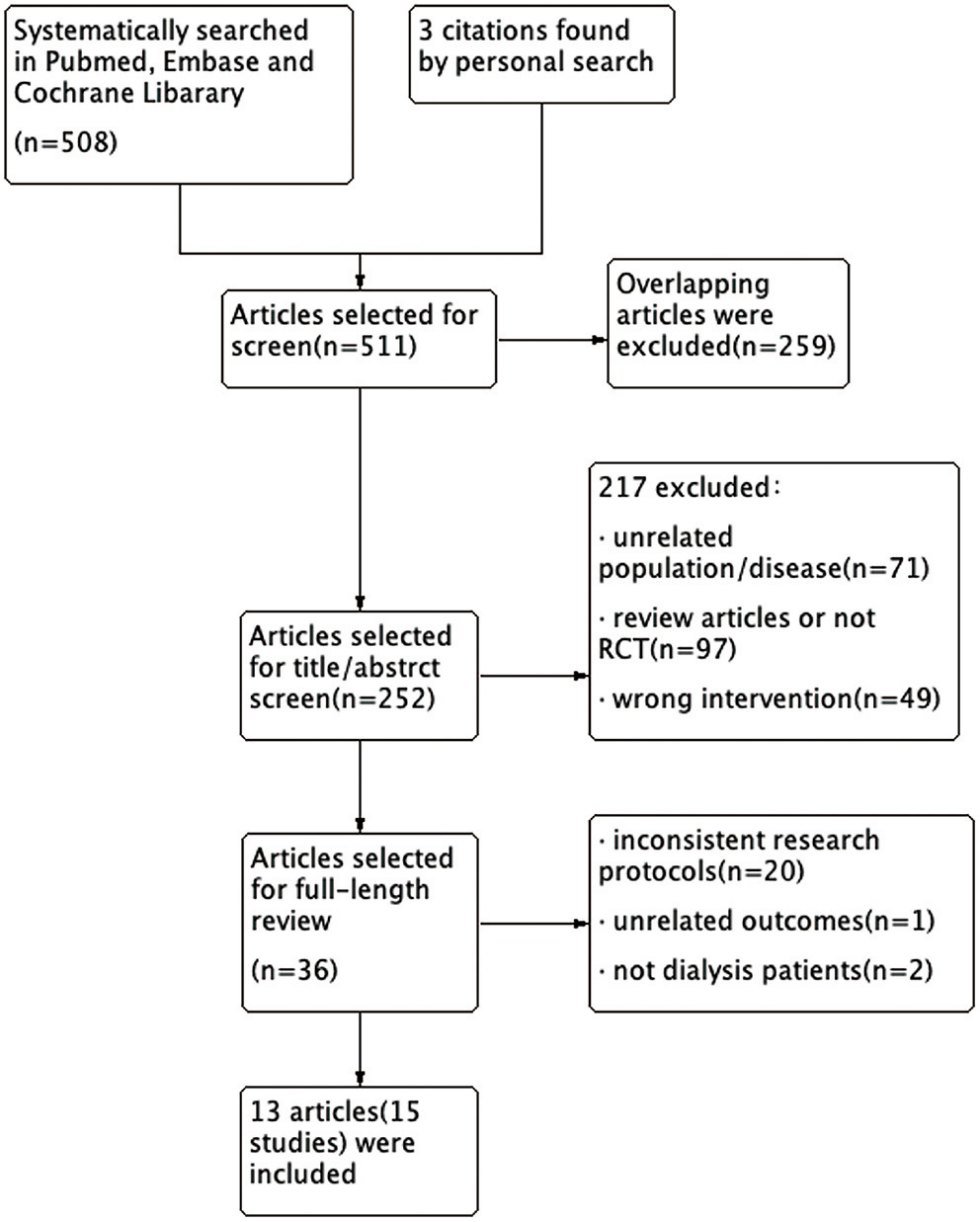
Flow diagram of the literature search process.

The process of study selection is schematically presented in the flowchart (Figure 1). Overall, the search terms identified 511 references. Of these, the reviewers excluded 259 articles from the initial screening. Subsequently, the majority of articles (217 articles) were excluded after reading the title and the abstract. After assessing the remaining 36 full text articles, we eliminated 23 additional articles since they failed to meet the inclusion criteria. Therefore, 15 randomized controlled trials (in 13 articles) were included in the meta-analysis.

### Search Characteristics and Quality

The studies enrolled 1,258 patients, with the mean age spanned 52.92 ± 6.90 to 70.45 ± 9.70 years. Of these articles, three were conducted in China (10, 15, 16), two in the United States (11, 17), two in Grace (9, 18), two in Japan (19, 20), two in Iran (21, 22), one in Brazil (23) and one in Chile (24). In terms of ethnicity, seven RCTs were performed in Asians and 6 articles (8 RCTs) included non-Asians. Nine RCTs were performed in patients on maintenance HD, 3 articles involved PD patients, and other 3 researches reported data in both HD and PD population (Table 1).

**Table 1:** Characteristics of enrolled researches.

**Study, year, country**	**Population characteristics**	**Spironolactone dosage**	**Dialysis**	**Duration**	**Outcomes**
Chaochao Wang (16); China	*N* = 96; Age (years) = 52.92 ± 6.91; Male (%) = 58.33 LVEF (%) = 39.78 ± 5.11; Dialysis vintage (months) = 32.32 ± 12.44	20 mg daily	PD	1 year	LVMI; LVEF
Charytan et al. (17); America	*N* = 102; Age (years) = 55.50 ± 12.00; Male (%) = 68.63 Hypertension (%) = 93.00; LVEF (%) = 68.11 ± 6.44 Dialysis vintage (years) = 3.4 (1.9–6.1)	25 mg; 50 mg daily	HD	9 months	Serum potassium; BP; LVMI; LVEF; ACM; CCV; side effects
Feniman (23); Brazil	*N* = 17; Age (years) = 54.12 ± 12.00; Male (%) = 52.94 LVEF (%) = 69.56 ± 4.06; Dialysis vintage (months) = 37.99 ± 58.26	25 mg daily	HD	6 months	Serum potassium; BP; LVMI; LVEF
Flevari et al. (9); Grace	*N* = 14; Age (years) = 59.5 ± 11.60; male (%) = 64.29 Hypertension (%) = 100% Dialysis vintage (years) = 2.4 ± 0.75	25 mg three times a week	HD	4 months	Serum potassium; BP; ACM; side effects
Gross et al. (11); America	*N* = 8; Age (years) = 53.00 ± 10.00; Male (%) = 37.50 Hypertension (%) = 37.50%	50 mg twice a week	HD	2 weeks	Serum potassium; BP
Hammer et al. (18); Grace	*N* = 97; Age (years) = 60.30 ± 13.20; Male (%) = 77.32 Hypertension (%) = 87.60; LVEF (%) = 59.76 ± 11.76 Dialysis vintage (months) = 42 (15.7–74.5)	50 mg daily	HD	10 months	BP; LVMI; LVEF; ACM; side effects
Ito et al. (19); Japan	*N* = 158; Age (years) = 56.49 ± 13.36; Male (%) = 71.52 LVEF (%) = 66.20 ± 10.31	25 mg Daily	PD	2 years	serum potassium; BP; LVMI; LVEF; ACM; CCV; side effects
Lin et al. (10); China	*N* = 253; Age (years) = 70.45 ± 9.70; Male (%) = 60.47 LVEF (%) = 57.75 ± 9.28; Dialysis vintage (months) = 42.70 ± 18.27	25 mg daily	HD/PD	2 years	Serum potassium; LVMI; LVEF; ACM; CCV; side effects
Matsumoto et al. (20); Japan	*N* = 309; Age (years) = 67.55 ± 11.75; Male (%) = 65.70 Hypertension (%) = 5.26; Dialysis vintage (months) = 113.58 ± 89.26	25 mg Daily	HD	3 years	ACM; CCV; side effects
Ni et al. (15); China	*N* = 76; Age (years) = 55.32 ± 13.15; Male (%) = 59.21 Hypertension (%) = 100; Dialysis vintage (months) = 55.74 ± 12.47	25 mg Daily	HD/PD	3 months	Serum potassium; BP; side effects
Taheri et al. (21); Iran	*N* = 16; Age (years) = 58.15 ± 7.88; Male (%) = 68.75 Hypertension (%) = 87.5; LVEF (%) = 32.5 ± 8.75	25 mg three times a week	HD	6 months	LVMI; LVEF; ACM; side effects
Taheri et al. (22); Iran	*N* = 18; Age (years) = 53.95 ± 15.31; Male (%) = 55.56 Hypertension (%) = 22.22; Dialysis vintage (months) = 44.05 ± 23.42	25 mg once every 2 days	PD	6 months	LVEF; ACM
Vukusich et al. (24); Chile	*N* = 53; Age (years) = 58.15 ± 5.06; Male (%) = 64.15 Hypertension (%) = 58.49; Dialysis vintage (years) = 8.29 ± 1.32	50 mg three times a week	HD	2 years	BP; ACM

*ACM, all-cause mortality; BP, blood pressure; CCV, cardiocerebrovascular diseases; HD, hemodialysis; LVEF, left ventricular ejection fraction; LVMI, left ventricular mass index; PD, peritoneal dialysis*.

**Figure 2:**
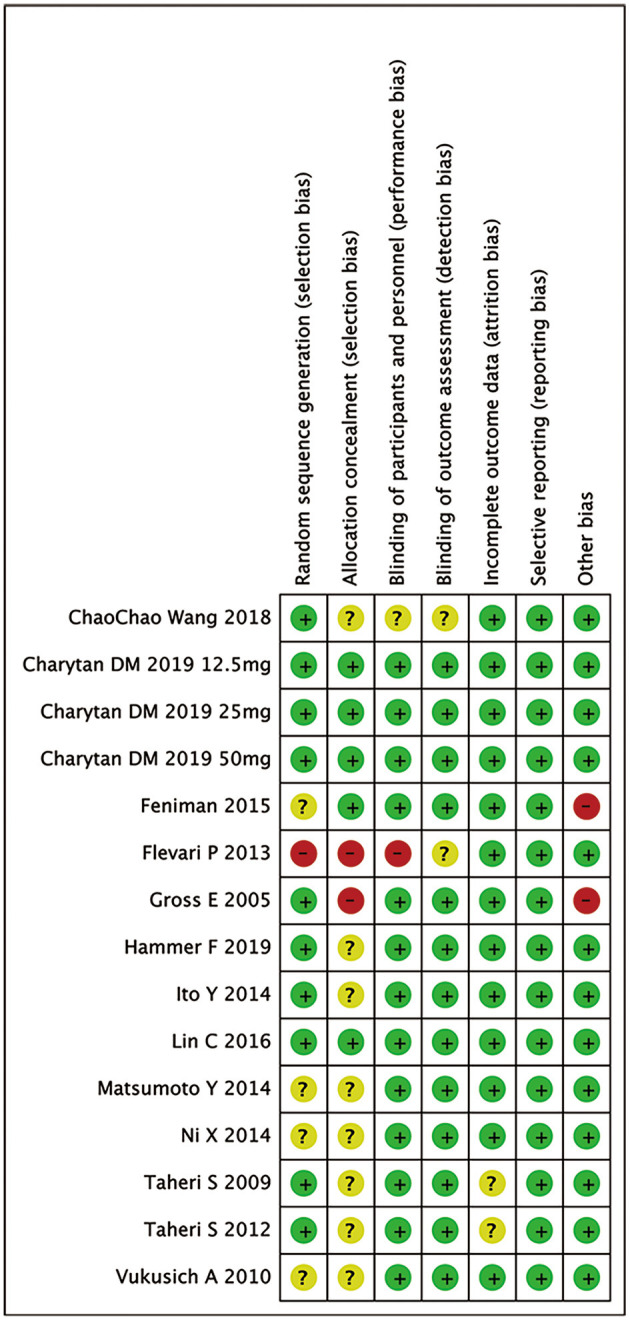
Risk of bias in analyzed studies. Unclear risk of bias: “?”, low risk of bias: “-”, and high risk of bias: “+”.

Figure 2 summarizes the risk of bias of 13 RCTs. Eight studies and 3 studies provided information of random sequence or allocation concealment, respectively. Eleven studies were triple-blinded and 1 had a single-blind design.

### Effects of Spironolactone on Primary Outcomes

#### ACM and CCV

**Figure 3:**
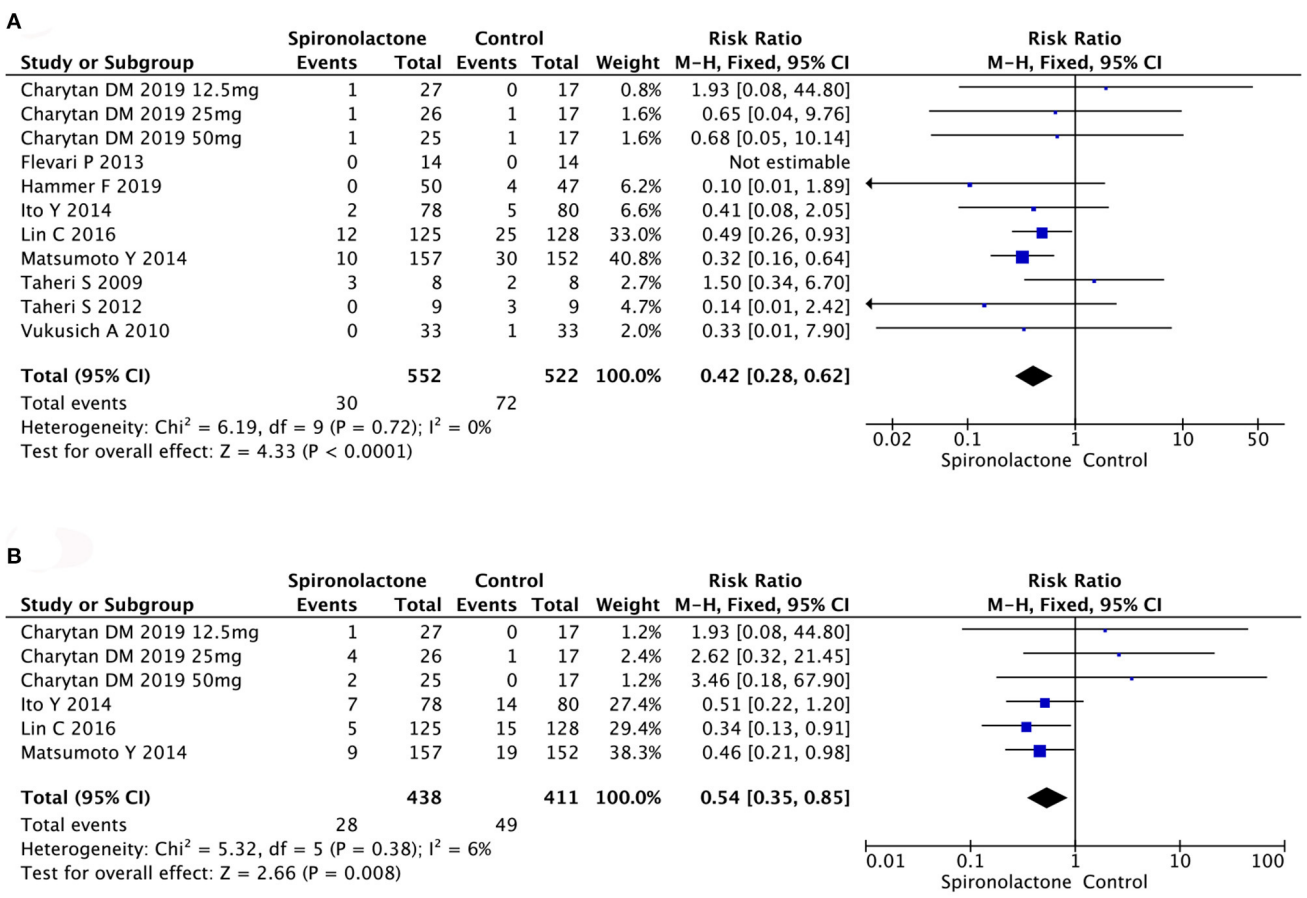
**(A)** Effect of spironolactone on ACM; **(B)** Effect of spironolactone on CCV. ACM, all-cause mortality; CCV, cardiocerebrovascular diseases.

Analysis of ACM in 1,074 patients in 11 studies found that spironolactone treatment significantly decreased ACM [RR = 0.42, 95%, CI = (0.28, 0.62), *P* < 0.0001]. Similarly, rate of CCV disease also decreased in spironolactone treatment group [RR = 0.54, 95%, CI = (0.35, 0.85), *P* = 0.008]. There were no heterogeneity (ACM: Chi^2^ = 6.19, *P* = 0.72, *I*^2^ = 0%; CCV: Chi^2^ = 5.32, *P* = 0.38, *I*^2^ = 6%) or publication bias (*P* = 0.770) (Figure 3).

#### Serum Potassium and Hyperkalemia

**Figure 4:**
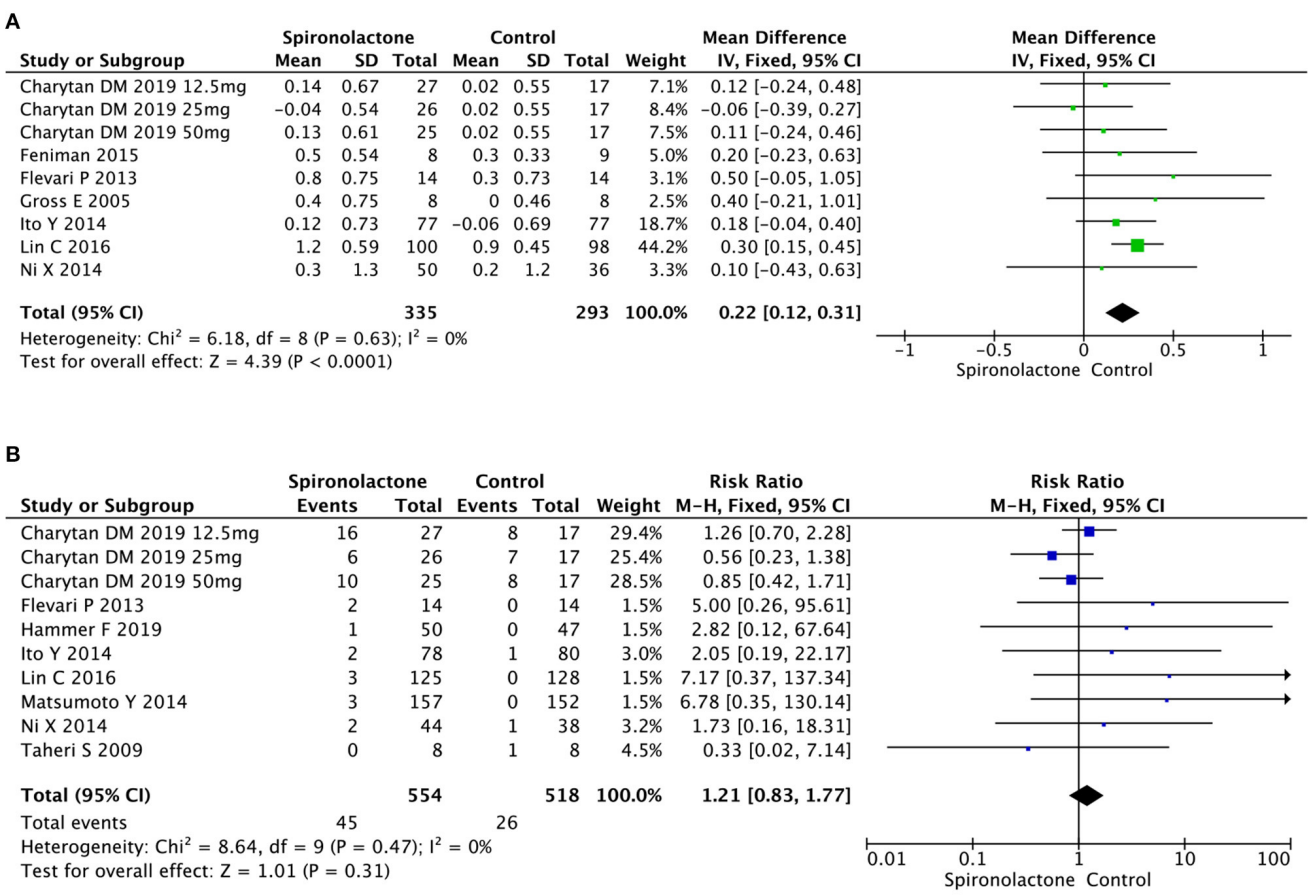
**(A)** Effect of spironolactone on serum potassium; **(B)** Effect of spironolactone on hyperkalemia.

As illustrated in Figure 4, meta-analysis showed patients in spironolactone group had significantly higher serum potassium level compared with control ones [MD = 0.22, 95%, CI = (0.12, 0.31), *P* < 0.0001, *I*^2^ = 0%]. However, incidence of hyperkalemia in 1,072 patients in 10 studies showed no significant difference between treated and untreated groups [RR = 1.21, 95%, CI = [0.83, 1.77], *P* = 0.31, *I*^2^ = 0%]. There was no publication bias (*P* = 0.119).

#### GYN

**Figure 5:**
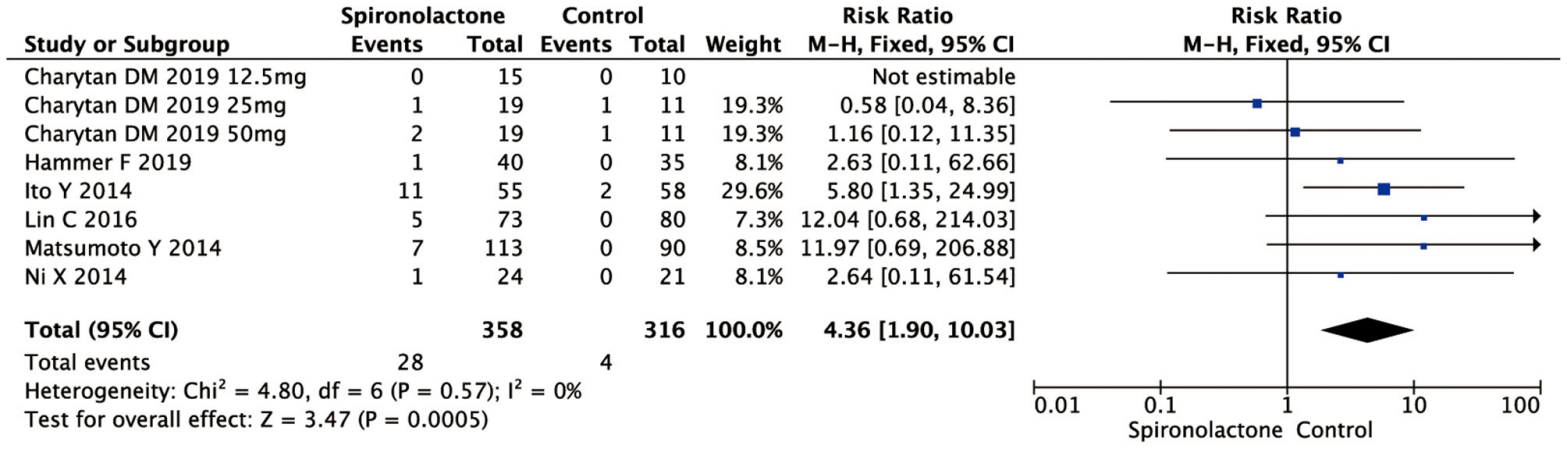
Effect of spironolactone on GYN. GYN, gynecomastia.

Analysis of the effects of spironolactone on GYN occurrence in 674 subjects revealed an increase in experimental vs. control groups [RR = 4.36, 95%, CI = (1.90, 10.03), *P* = 0.0005], with no heterogeneity (Chi^2^ = 4.80, *P* = 0.57, *I*^2^ = 0%) (Figure 5).

### Effects of Spironolactone on the Secondary Outcomes

#### Blood Pressure

**Figure 6:**
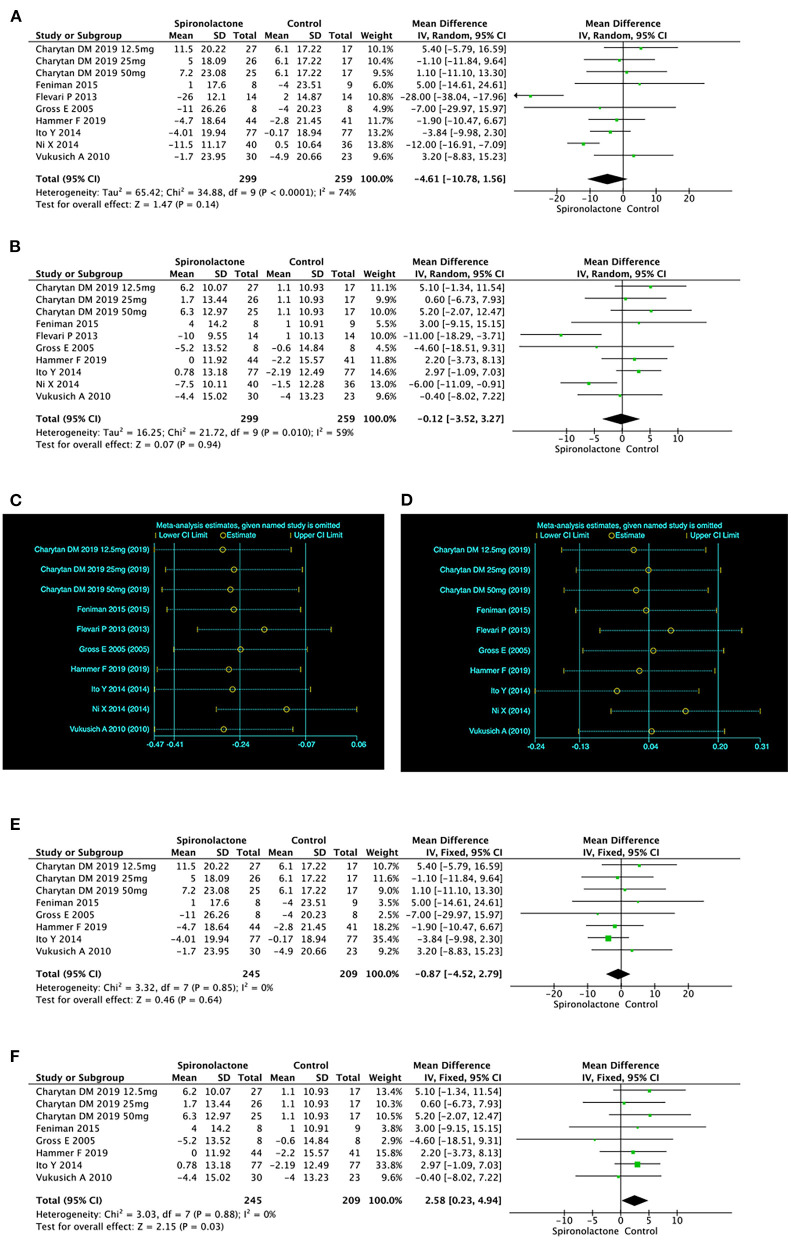
**(A)** Effect of spironolactone on SBP; **(B)** Effect of spironolactone on DBP; **(C)** Sensitive analysis of SBP; **(D)** Sensitive analysis of DBP; **(E)** Effect of spironolactone on SBP after sensitive analysis; **(F)** Effect of spironolactone on DBP after sensitive analysis. DBP, diastolic blood pressure; SBP, systolic blood pressure.

The pooled analysis of 10 researches showed that there was no significant difference in SBP [MD = −4.61, 95%, CI = (−10.78, 1.56), *P* = 0.14, *I*^2^ = 74%] or DBP [MD = −0.12, 95%CI = (−3.52, 3.27), *P* = 0.94, *I*^2^ = 59%]between two groups. The considerable heterogeneity was not linked to the country, treatment duration or type of renal replacement (Figures 6A,B). However, sensitivity analysis showed that the results of Flevari et al. (9) and Ni et al. (15) differed significantly from other studies, leading to unstable meta-analysis results for SBP and DBP (Figures 6C–F).

#### Heart Function

**Figure 7:**
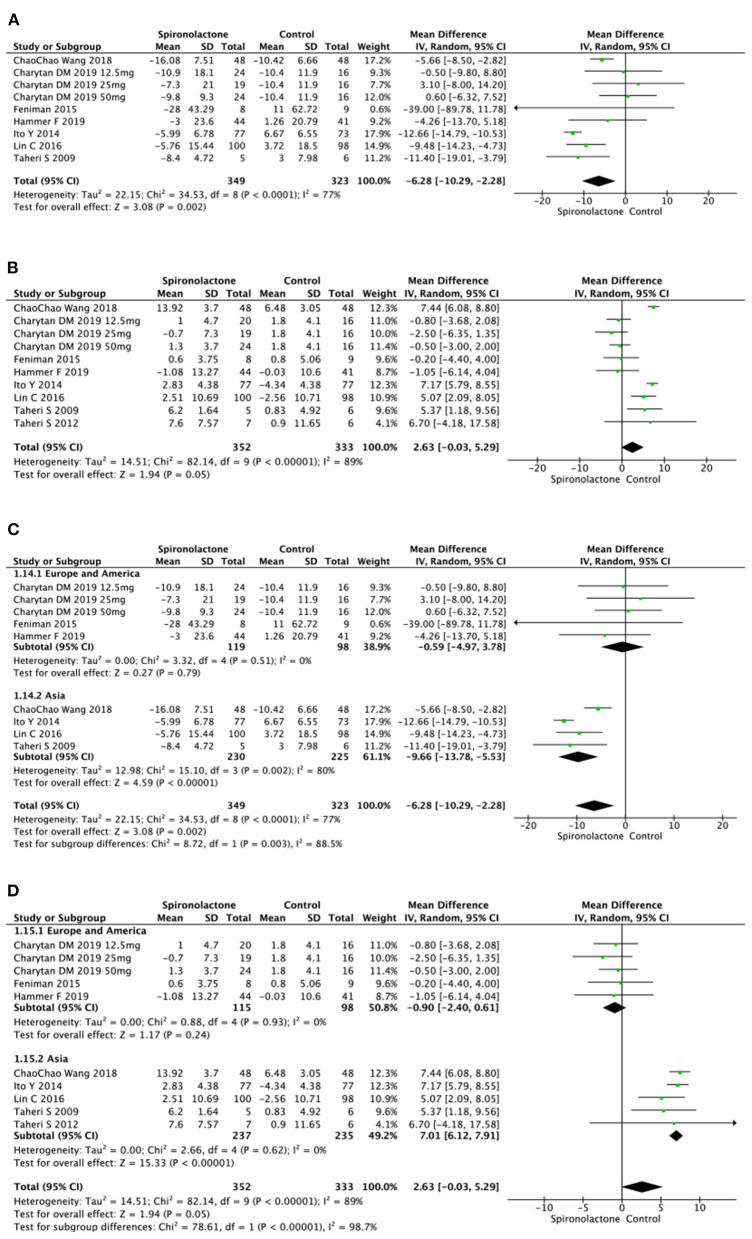
**(A)** Effect of spironolactone on LVMI; **(B)** Effect of spironolactone on LVEF; **(C)** Effect of spironolactone on LVMI after subgroup analysis; **(D)** Effect of spironolactone on LVEF after subgroup analysis. LVEF, left ventricular ejection fraction; LVMI, left ventricular mass index.

Available data of heart function included LVMI and LVEF. Data on LVMI and LVEF were respectively reported in several trials, and results indicated a significant decrease of LVMI [MD = −6.28, 95%, CI = (−10.29, −2.28), *P* = 0.002, *I*^2^ = 77%] between two groups. However there was no significant difference in LVEF (MD = 2.63, 95%, CI = (−0.03, 5.29), *P* = 0.05, *I*^2^ = 89%] in treated patients vs. untreated ones. Further subgroup analysis was conducted due to the non-ignorable heterogeneity. The data on LVMI showed that spironolactone was beneficial for Asian patients [MD = −9.66, 95%, CI= (−13.78, −5.53), *P* < 0.00001, *I*^2^ = 80%], while there was no significant difference between patients with and without spironolactone in Europe and the United States subgroup [MD = −0.59, 95%, CI = (−4.97, 3.78), *P* = 0.79, *I*^2^ = 0%]. Similarly, the benefit of spironolactone in increasing LVEF only existed in Asian patients (MD = 7.01, 95%, CI= (6.12, 7.91), *P* < 0.00001, *I*^2^ = 0%), not European or American subjects [MD = −0.90, 95%, CI = [−2.40, 0.61], *P* = 0.24, *I*^2^ = 0%] (Figure 7).

### Subgroup Analysis

**Figure 8:**
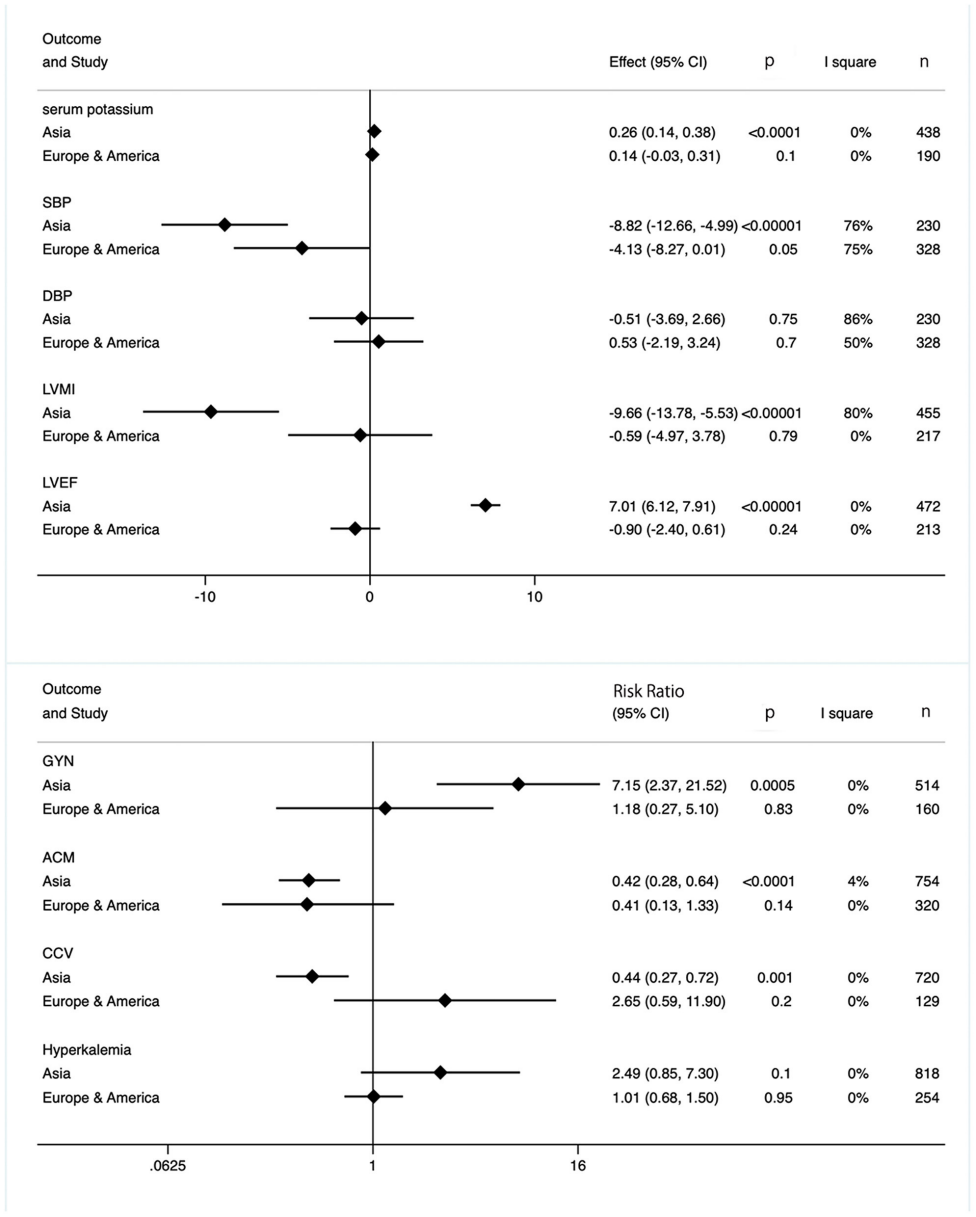
Subgroup analysis based on countries.

Further subgroup analysis based on country was performed. In Asian subgroup, the pooled analysis found significant difference in serum potassium level, SBP, ACM, LVMI, LVEF, and incidence of CCV and GYN between experimental and control patients. Besides, spironolactone supplementation did not cause a consistent change in DBP and hyperkalemia occurrence. However, in European and American subjects, meta-analysis showed that above mentioned indexes did not confer difference after spironolactone application (Figure 8).

**Figure 9:**
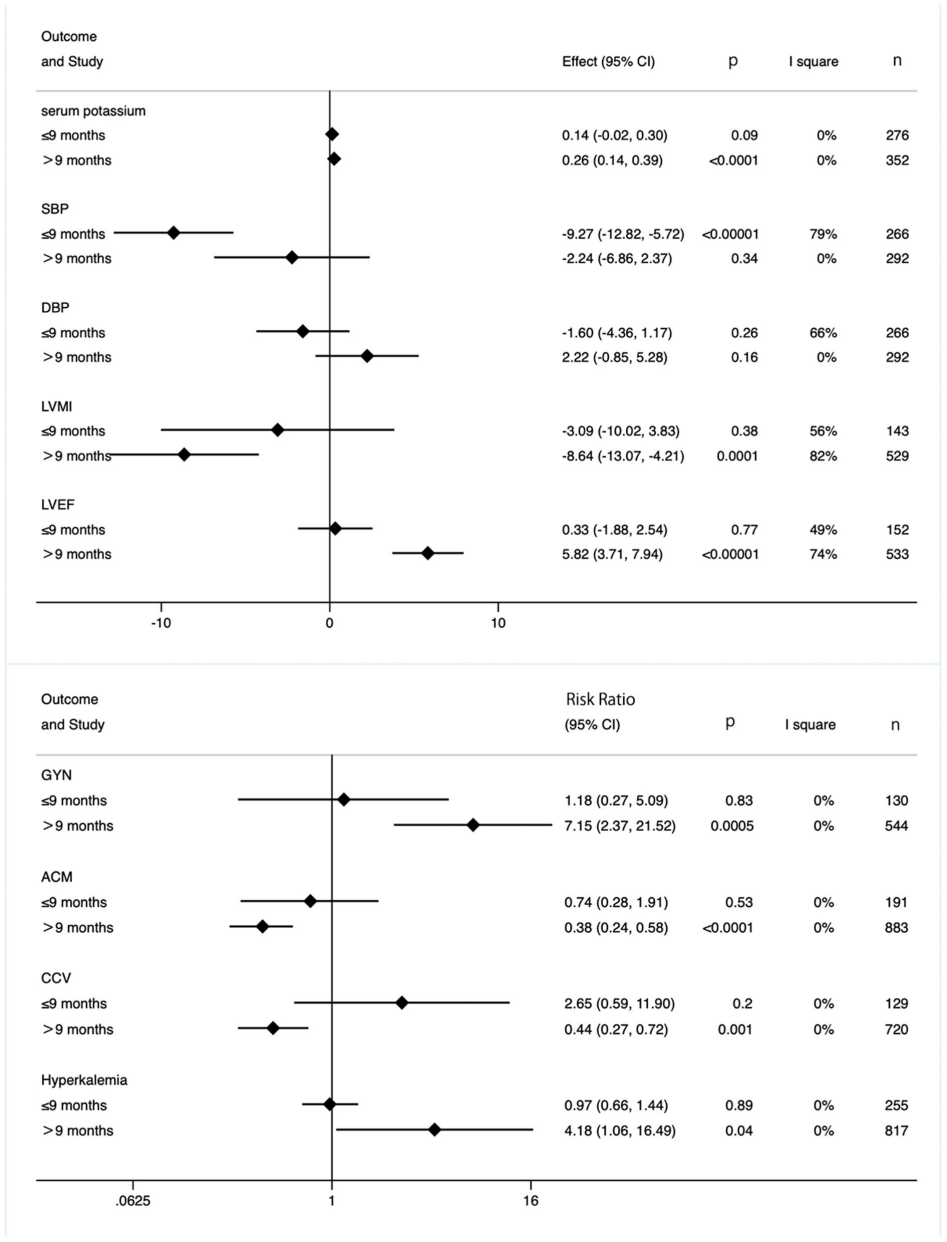
Subgroup analysis based on duration.

To eliminate the effect of duration, we also took subgroup analysis based on duration period. As shown in Figure 9, although longer intervention times (>9 months) had significant benefits on cardiac function (lower ACM and CCV occurrence, decreased LVMI, and increased LVEF), side effects (GYN, hyperkalemia, serum potassium) were significantly increased. However, in the subgroup with a treatment duration of less than or equal to 9 months, neither cardiac-related efficacy nor side effects were significant (Figure 9).

## Discussion

In this meta-analysis of 15 RCTs of 1,258 dialysis-dependent patients, we suggested that spironolactone treatment affected various parameters associated with cardiovascular system. Spironolactone decreased ACM, SBP, LVMI. Although the serum potassium level significantly increased, spironolactone did not elevate the occurrence of hyperkalemia. The further subgroup analysis implied the intergroup differences across countries. Spironolactone treatment had more obvious efficacy (decreased ACM, CCV, LVMI, and SBP, also increased LVEF) and more severe adverse effects (increased GYN occurrence and serum potassium) on Asian patients. However, spironolactone did not affect these markers in European and American population. Seven RCTs used spironolactone for more than 9 months, appearing more effective in reducing ACM and CCM as well as increasing incidence of GYN and hyperkalemia than those RCTs with shorter application duration.

Hypertension often exists in patients with ESRD, accompanied by activated RASS and secondary hyperaldosteronemia (25). Aldosterone adversely affects blood pressure through both cellular and nervous mechanism. In blood vessels, mineralocorticoid not only decreases deformability of endothelial cells (26), but also suppresses bioactivity of nitric oxide in smooth muscle cells (27), therefore regulates blood pressure. Meanwhile, aldosterone receptors in the central nervous system may enhance sympathetic activity and thus contribute to hypertension (28). Although our meta-analysis didn‘t find antihypertensive effect of spironolactone, the high heterogeneity promoted us to perform sensitive analysis, which found data provided by Ni et al. (15) and Flevari et al. (9) were the source of heterogeneity. It may because all subjects included in these two studies were hypertensive, while other researches had a lower proportion of participants with hypertension (37.5 to 87.6%). Patients with hypertension, especially resistant hypertension, may be particularly prone to salt and water retention, therefore, more susceptible to spironolactone (29). Abnormal level of serum aldosterone promotes the production of profibrotic TGF-βsignaling and following cardiac fibrosis (30). Thus, using aldosterone antagonist can relief cardiac fibrosis in nephrectomized animals (31, 32). Similarly, we also observed rate of LVEF and LVMI significantly changed after MRAs treatment. Spironolactone may also benefit CCV system through following pathways: (1) blocking aldosterone effect on collagen formation, therefore, inhibiting left ventricle remodeling (33); (2) the antihypertensive effect of spironolactone improves vascular endothelial function (34); (3) preventing peritoneal inflammation and fibrosis thereby maintaining peritoneal function (35).

Our research also noted the safety of spironolactone in dialysis-dependent patients, with spironolactone treatment showing a tendency to increase serum potassium concentration, but an unchanged incidence of hyperkalemia. Hyperkalemia, which is well-recognized as adverse effect of MRAs, always prevent the physician from applying spironolactone. Notably, in our pooled-analysis, most of patients who dropped out of the study because of hyperkalemia were hemodialysis (HD) patients, not PD patients. These results are similar to previous findings showing the greater removal of potassium in PD than HD subjects (36, 37). Since dialysis patients are more dependent on dialysis rather than kidney to excrete potassium, using spironolactone maybe safer in dialysis patients than in non-dialysis ones. GYN also appeared with spironolactone application (38), and a significant occurrence of GYN was noted in treated group. The risk of GYN may be minimized if patients used low dose of spironolactone or switched to selective MRAs, such as eplerenone (19).

In subgroup analysis, the effects, including efficacy and side effects, of spironolactone were more significant in Asian patients. Actually, this kind of racial difference existed in several clinical studies on MRAs. Vardeny et al. reported that non-African Americans might have greater beneficial effect from spironolactone supplement than African Americans (39). Besides the unclear genetic mechanism of racial disparity, different selection of ACEI or ARB by ethnic groups may also cause inter-subgroup heterogeneity. The high incidence of cough among Asian patients makes them prone to choose ARB rather than ACEI (40, 41), which may contribute to different results on efficacy and side effects between Asian and non-Asian subgroups (42, 43). Besides, relatively longer intervention duration in Asian populations (3 ~ 36 months) than in European and American ones (0.5 ~ 24 months) should also be taken into account. Till now, the safe duration of spironolactone treatment is still controversial. Spironolactone exerts cardio-protective effects by inhibiting MRs on the one hand and reduces potassium excretion owing to Na^+^/K^+^ pump inhibition on the other hand. These double-edged sword effects do not occur simultaneously. Spironolactone elevates serum potassium at an early stage, while cardioprotective effects appear later (44), suggesting close laboratory surveillance for patients newly initiating therapy with MRAs. From this pooled-analysis, longer duration (more than 9 months) was related to an increased LVEF, decreased LVMI, ACM, and CCV, however, the incidence of GYN and ACM also raised. We also did further subgroup-analysis based on dosage, and surprisedly, the results indicated that the lower dosage (≤25 mg) had more obvious side effect and efficacy than higher dosage (50 mg) (Supplementary Figure 2). This may due to the possibility that Asians are more sensitive to drugs and therefore, based on former experience, all Asian groups have chosen smaller doses. Thus, optimal dosage in terms of safety and efficacy remains a critical question that needs to be addressed. Epidemiological evidence suggests that diabetes mellitus is one of the most common modifiable risk factors for CCV and ACM (45). However, all but one article [the study by Vukusich et al. (24)] included only non-diabetic patients, other 12 articles included diabetic and non-diabetic patients. Thus, we failed to perform a diabetes-based subgroup analysis. Besides, as an early sensitive indicator, carotid intima-media thickness has been used as a surrogate endpoint for CCV and ACM to access the efficacy of certain interventions in the past few years (46). Therefore, further studies limited to non-diabetic patients with reported data on carotid intima-media thickness are recommended.

The strength of this study is the strict selection range that only includes RCTs. Meanwhile, without the restrictions of follow-up duration and renal replacement type, we maximized the collected information while selection bias or other potential bias were minimized. Moreover, to the best of our knowledge, this is the first meta-analysis to provide evidence of racial differences in spironolactone use in dialysis-dependent patients. Our results should also be interpreted within the context of several limitations. These include the relatively small sample size and various duration of treatment period. Since spironolactone is an MRA, future studies should report serum as well as urine aldosterone level. Most RCTs included are single-center trials. However, the racial factor is one of the sources of intergroup heterogeneity, thus, multi-central research with patients from various continents is needed in following studies. Moreover, Ito et al. (19) proposed inconsistent changes in kidney-related indicators after spironolactone use in men and women, suggesting further researches to report outcomes according to gender.

## Conclusions

Patients undergoing dialysis can achieve cardiac benefit (LVEF, LVMI, CCV, and ACM) after spironolactone treatment, while the risk of GYN and serum potassium concentration also increased. However, spironolactone does not affect the incidence rate of hyperkalemia. Asian patients can achieve more obvious benefit, although more side effects, from spironolactone than European and American subjects. The use of spironolactone for more than 9 months can have a pharmacological effect compared to a shorter course of treatment. Studies with larger scales and multi-countries are advocated to further evaluate the balance of efficacy and adverse effects in spironolactone use.

## Data Availability Statement

The original contributions presented in the study are included in the article/Supplementary Material, further inquiries can be directed to the corresponding author.

## Author Contributions

JL assisted in conceptualization, data curation, formal analysis, investigation, and writing—original draft preparation. WJ assisted in conceptualization, formal analysis, and writing—original draft preparation. CY assisted in resources, writing—review and editing, and supervision. All authors contributed to the article and approved the submitted version.

## Funding

This work was financially supported through grants from the National Natural Science Foundation of China (Nos. 81873609, 82170696, and 81800631), Shanghai Sailing Program (19YF1444200 and 19YF1444400), and Tongji Hospital Medical Records Program (TJ (DB)2103).

## Conflict of Interest

The authors declare that the research was conducted in the absence of any commercial or financial relationships that could be construed as a potential conflict of interest.

## Publisher's Note

All claims expressed in this article are solely those of the authors and do not necessarily represent those of their affiliated organizations, or those of the publisher, the editors and the reviewers. Any product that may be evaluated in this article, or claim that may be made by its manufacturer, is not guaranteed or endorsed by the publisher.

## Supplementary Material

Supplementary Figure 1 Graphic abstract.

Supplementary Figure 2 Subgroup analysis based on dosage.
