# Supplementary figures and images for: Safety and Efficacy of Spironolactone in Dialysis-Dependent Patients: Meta-Analysis of Randomized Controlled Trials
Source: Front Med (Lausanne). 2022 Mar 17;9:828189. doi: 10.3389/fmed.2022.828189 (PMC8970057; doi:10.3389/fmed.2022.828189)

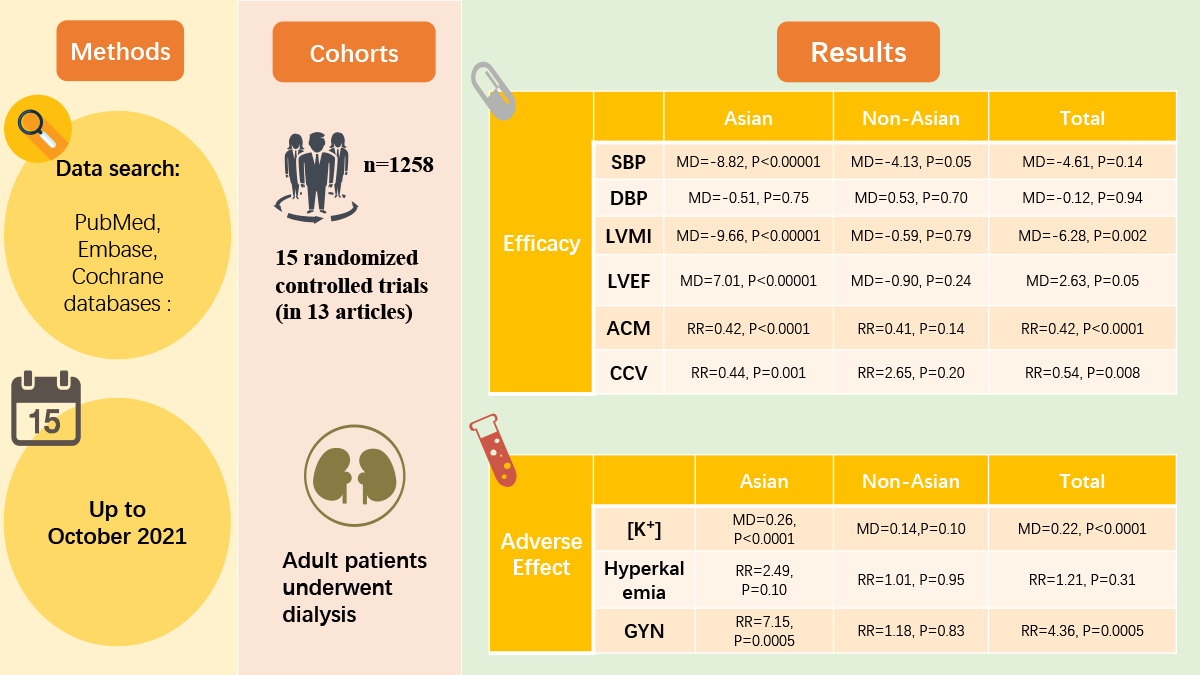

Supplement: Supplementary Figure 1 — Graphic abstract. [file Image_1.TIF]

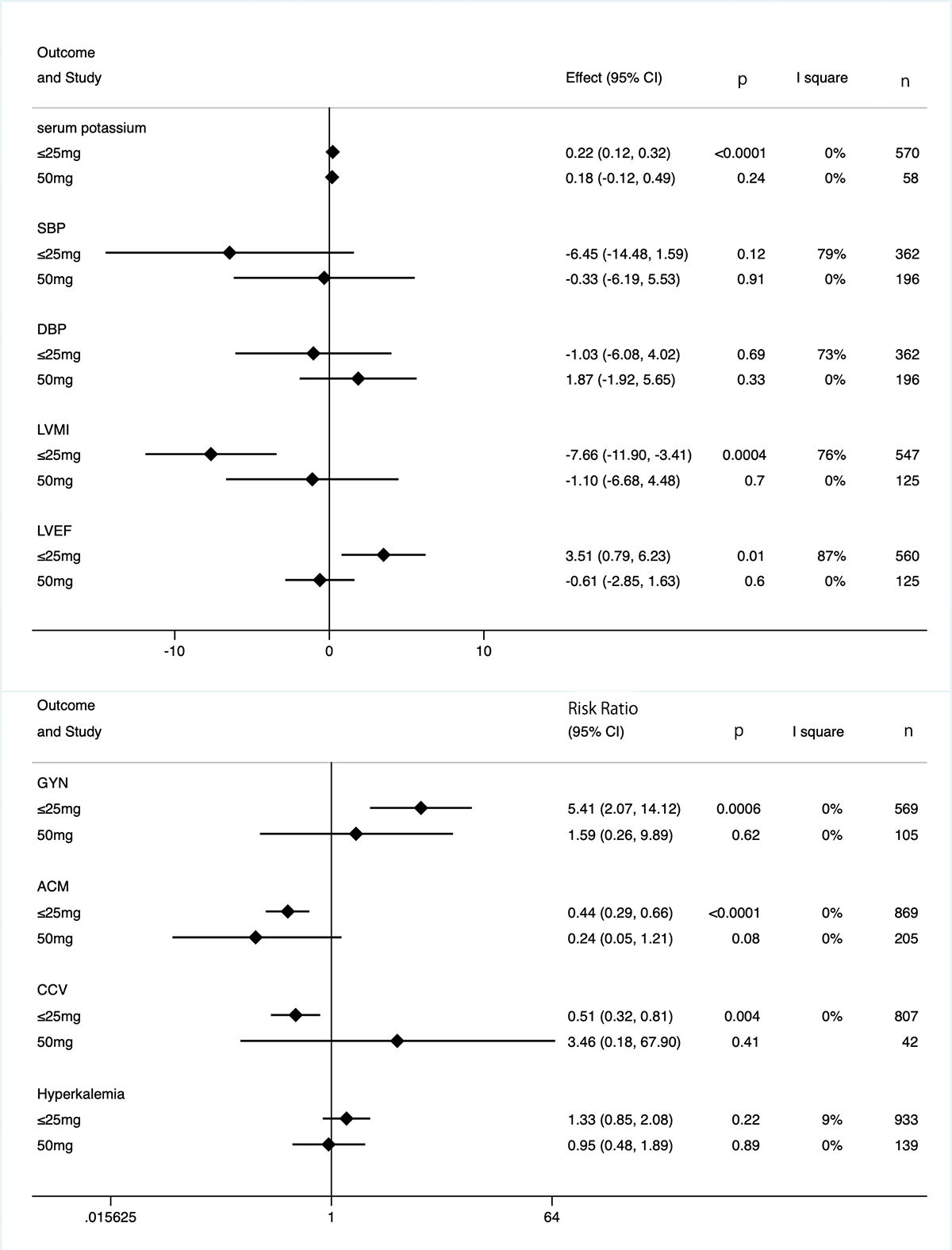

Supplement: Supplementary Figure 2 — Subgroup analysis based on dosage. [file Image_2.TIF]
