# Supplementary material for: Safety and Efficacy of Spironolactone in Dialysis-Dependent Patients: Meta-Analysis of Randomized Controlled Trials
Source: Front Med (Lausanne). 2022 Mar 17;9:828189. doi: 10.3389/fmed.2022.828189 (PMC8970057; doi:10.3389/fmed.2022.828189)
Supplement: Supplementary file 3 [file Table_1.DOC]

Appendix A: Search strategies

Embase

1. exp kidney failure/
2. (renal adj2 insufficien*).mp.
3. (kidney* adj2 insufficien*).mp.
4. (chronic adj2 renal).mp.
5. (renal* adj2 fail*).mp.
6. (kidney* adj2 fail*).mp.
7. (end-stage adj2 kidney).mp.
8. (end-stage adj2 renal).mp.
9. esrd.tw.
10. ckd.tw.
11. crd.tw.
12. ckf.tw.
13. crf.tw.
14. or/1-13
15. exp renal replacement therapy/
16. dialyses.mp.
17. dialysis.mp.
18. hemodialyses.mp.
19. haemodialyses.mp.
20. haemodialysis.mp.
21. hemodialysis.mp.
22. or/15-21
23. 14 and 22
24. exp spironolactone/
25. spirolactone.mp.
26. veroshpiron.mp.
27. Spirobeta.mp.
28. Spirogamma.mp.
29. Spirolang.mp.
30. Spironoisis.mp.
31. Spirono Isis.mp.
32. Spironone.mp.
33. Spirospare.mp.
34. Aldactone.mp.
35. Aquareduct.mp.
36. Duraspiron.mp.
37. Espironolactona Alter.mp.
38. Espironolactona Mundogen.mp.
39. Flumach.mp.
40. Frumikal.mp.
41. Jenaspiron.mp.
42. Novo-Spiroton.mp.
43. Novo Spiroton.mp.
44. NovoSpiroton.mp.
45. Practon.mp.
46. SC-9420.mp.
47. SC 9420.mp.
48. SC9420.mp.
49. Spiro L.U.T. .mp.
50. Spiro Von Ct.mp.
51. Ct, Spiro Von.mp.
52. Von Ct, Spiro.mp.
53. or/24-52
54. 23 and 53
55. Clinical trial/
56. Randomized controlled trial/ 77 Randomization/
57. Placebo/
58. Randomi?ed controlled trial$.tw.
59. Rct.tw.
60. Random allocation.tw.
61. Randomly allocated.tw.
62. Allocated randomly.tw.
63. (allocated adj2 random).tw.
64. Double blind$.tw.
65. Prospective study/
66. or/55-65
67. Case study/
68. Case report.tw.
69. Abstract report/ or letter/
70. or/67-69
71. 66 not 70
72. 54 and 71

MEDLINE 检索词：

1. exp Renal Insufficiency, Chronic/
2. (renal adj2 insufficien*).mp.
3. (kidney* adj2 insufficien*).mp.
4. (chronic adj2 renal).mp.
5. (renal* adj2 fail*).mp.
6. (kidney* adj2 fail*).mp.
7. (end-stage adj2 kidney).mp.
8. (end-stage adj2 renal).mp.
9. esrd.tw.
10. ckd.tw.
11. crd.tw.
12. ckf.tw.
13. crf.tw.
14. or/1-13
15. exp Renal Dialysis/
16. dialyses.mp.
17. dialysis.mp.
18. hemodialyses.mp.
19. haemodialysis.mp.
20. haemodialyses.mp.
21. hemodialysis.mp.
22. or/15-21
23. 14 and 22
24. exp spironolactone/
25. spirolactone.mp.
26. veroshpiron.mp.
27. Spirobeta.mp.
28. Spirogamma.mp.
29. Spirolang.mp.
30. Spironoisis.mp.
31. Spirono Isis.mp.
32. Spironone.mp.
33. Spirospare.mp.
34. Aldactone.mp.
35. Aquareduct.mp.
36. Duraspiron.mp.
37. Espironolactona Alter.mp.
38. Espironolactona Mundogen.mp.
39. Flumach.mp.
40. Frumikal.mp.
41. Jenaspiron.mp.
42. Novo-Spiroton.mp.
43. Novo Spiroton.mp.
44. NovoSpiroton.mp.
45. Practon.mp.
46. SC-9420.mp.
47. SC 9420.mp.
48. SC9420.mp.
49. Spiro L.U.T. .mp.
50. Spiro Von Ct.mp.
51. Ct, Spiro Von.mp.
52. Von Ct, Spiro.mp.
53. or/24-52
54. 23 and 53
55. Randomized Controlled Trials as Topic/
56. randomized controlled trial/
57. Random Allocation/
58. Double Blind Method/
59. clinical trial/
60. clinical trial, phase i.pt.
61. clinical trial, phase ii.pt.
62. clinical trial, phase iii.pt.
63. clinical trial, phase iv.pt.
64. randomized controlled trial.pt.
65. multicenter study.pt.
66. clinical trial.pt.
67. exp Clinical Trials as topic/
68. or/55-67
69. (clinical adj trial$).tw.
70. ((singl$ or doubl$ or treb$ or tripl$) adj (blind$3 or mask$3)).tw.
71. randomly allocated.tw.
72. (allocated adj2 random$).tw.
73. or/69-72
74. 68 or 73
75. case report.tw.
76. letter/
77. historical article/
78. or/75-77
79. 74 not 78
80. 54 and 79

**Pubmed:**

(((((((((((((((((((((((((((((((((("Spironolactone"[Mesh]) OR ("Spirolactone"[Title/Abstract])) OR ("Veroshpiron"[Title/Abstract])) OR ("Verospirone"[Title/Abstract])) OR ("Spiractin"[Title/Abstract])) OR ("Spirobeta"[Title/Abstract])) OR ("Spirogamma"[Title/Abstract])) OR ("Spirolang"[Title/Abstract])) OR ("Spirono-Isis"[Title/Abstract])) OR ("Spirono Isis"[Title/Abstract])) OR ("Spironone"[Title/Abstract])) OR ("Spirospare"[Title/Abstract])) OR ("Aldactone"[Title/Abstract])) OR ("Verospiron"[Title/Abstract])) OR ("Aldactone A"[Title/Abstract])) OR ("Aquareduct"[Title/Abstract])) OR ("Duraspiron"[Title/Abstract])) OR ("Espironolactona Alter"[Title/Abstract])) OR ("Espironolactona Mundogen"[Title/Abstract])) OR ("Flumach"[Title/Abstract])) OR ("Frumikal"[Title/Abstract])) OR ("Jenaspiron"[Title/Abstract])) OR ("Novo-Spiroton"[Title/Abstract])) OR ("Novo Spiroton"[Title/Abstract])) OR ("NovoSpiroton"[Title/Abstract])) OR ("Practon"[Title/Abstract])) OR ("SC-9420"[Title/Abstract])) OR ("SC 9420"[Title/Abstract])) OR ("SC9420"[Title/Abstract])) OR ("Spiro L.U.T."[Title/Abstract])) OR ("Spiro Von Ct"[Title/Abstract])) OR ("Ct, Spiro Von"[Title/Abstract])) OR ("Von Ct, Spiro"[Title/Abstract]) AND (clinicaltrial[Filter])) AND ((((((((((((((((("Kidney Failure, Chronic"[Mesh]) OR ("End-Stage Kidney Disease"[Title/Abstract])) OR ("Disease, End-Stage Kidney"[Title/Abstract])) OR ("End Stage Kidney Disease"[Title/Abstract])) OR ("Kidney Disease, End-Stage"[Title/Abstract])) OR ("Chronic Kidney Failure"[Title/Abstract])) OR ("End-Stage Renal Disease"[Title/Abstract])) OR ("Disease, End-Stage Renal"[Title/Abstract])) OR ("End Stage Renal Disease"[Title/Abstract])) OR ("Renal Disease, End-Stage"[Title/Abstract])) OR ("Renal Disease, End Stage"[Title/Abstract])) OR ("Renal Failure, End-Stage"[Title/Abstract])) OR ("End-Stage Renal Failure"[Title/Abstract])) OR ("Renal Failure, End Stage"[Title/Abstract])) OR ("Renal Failure, Chronic"[Title/Abstract])) OR ("Chronic Renal Failure"[Title/Abstract])) OR ("ESRD"[Title/Abstract]) AND (clinicaltrial[Filter]))) AND ((Randomized Controlled Trial [Publication Type]) OR ("RCT"[Title/Abstract]) AND (clinicaltrial[Filter]))
